# Whole-body magnetic resonance imaging: technique, guidelines and key applications

**DOI:** 10.3332/ecancer.2021.1164

**Published:** 2021-01-07

**Authors:** Paul Summers, Giulia Saia, Alberto Colombo, Paola Pricolo, Fabio Zugni, Sarah Alessi, Giulia Marvaso, Barbara Alicja Jereczek-Fossa, Massimo Bellomi, Giuseppe Petralia

**Affiliations:** 1 Division of Radiology, IEO European Institute of Oncology IRCCS, 20141 Milan, Italy; 2 Advanced Screening Centers, ASC Italia, 24060 Castelli Calepio, Bergamo, Italy; 3 Division of Radiotherapy, IEO European Institute of Oncology IRCCS, 20141 Milan, Italy; 4 Department of Oncology and Hemato-Oncology, University of Milan, 20122 Milan, Italy; 5 Precision Imaging and Research Unit, Department of Medical Imaging and Radiation Sciences, IEO European Institute of Oncology IRCCS, 20141 Milan, Italy

**Keywords:** magnetic resonance imaging, whole-body, diffusion-weighted imaging, oncology

## Abstract

Whole-body magnetic resonance imaging (WB-MRI) is an imaging method without ionising radiation that can provide WB coverage with a core protocol of essential imaging contrasts in less than 40 minutes, and it can be complemented with sequences to evaluate specific body regions as needed. In many cases, WB-MRI surpasses bone scintigraphy and computed tomography in detecting and characterising lesions, evaluating their response to therapy and in screening of high-risk patients. Consequently, international guidelines now recommend the use of WB-MRI in the management of patients with multiple myeloma, prostate cancer, melanoma and individuals with certain cancer predisposition syndromes. The use of WB-MRI is also growing for metastatic breast cancer, ovarian cancer and lymphoma as well as for cancer screening amongst the general population. In light of the increasing interest from clinicians and patients in WB-MRI as a radiation-free technique for guiding the management of cancer and for cancer screening, we review its technical basis, current international guidelines for its use and key applications.

## Background

The introduction of multiple receiver coils and table movement for multi-station scanning in the late 1990s transformed magnetic resonance imaging (MRI) from a segmental to a whole-body (WB) imaging modality [1–3]. Progressive improvements in scanner homogeneity and gradient systems facilitated the introduction of diffusion-weighted imaging (DWI) [4] WB examinations by Takahara *et al* [5]. Evidence that DWI provides good diagnostic performance in the detection, characterisation and monitoring of therapy of many cancers, consolidated in a first consensus meeting of experts in 2009 [6], has led to DWI having a central role in WB-MRI. Further developments, including accelerated imaging and improved sequence design, have brought improvements in image quality and rendered WB-MRI feasible in a clinically acceptable scan-time of under 40 minutes, opening further opportunities for clinical use [7–9]. In the course of this progression, WB-MRI has become an established part of the management of several cancer histotypes [10–15]. In light of the increasing interest from clinicians and patients in WB-MRI as a radiation-free technique for guiding the management of cancer and for cancer screening, we review its technical basis, current international guidelines for its use and key applications.

## Technical considerations

### Sequences and image acquisition

WB-MRI imaging protocols generally include morphological T1- and T2-weighted sequences along with DWI (Table 1). T1-weighted images are usually obtained with gradient-echo (GRE) Dixon acquisitions [16], producing in- and opposed-phase images that allow the calculation of fat-only and water-only images. These are useful in the detection, characterisation and response assessment of bone metastases. As an alternative, a T1-weighted 3D turbo spin echo (TSE) sequence could be performed [17], but this typically results in a longer acquisition and foregoes the discrimination of water and fat components.

T2-weighted images, acquired using single-shot or half-acquisition turbo spin echo (HASTE) sequences without fat suppression, can be used for the evaluation of disease in organs other than bone [18] with a good trade-off between duration and signal to noise ratio (SNR) [19], and can be helpful in confirming the presence of spinal cord compression [20].

Sagittal T1-weighted TSE and fat-saturated (short tau inversion recovery—STIR) T2-weighted TSE images of the whole spine are used for the detection of vertebral metastases, fractures and spinal cord compression [11, 13].

The final essential component of a WB-MRI protocol is a single-shot diffusion-weighted echo-planar imaging sequence. DWI has become central to WB-MRI due to its ability to detect malignant lesions characterised by high cellularity. Acquiring multiple averages of the DWI data during free-breathing is recommended in order to reduce motion artefacts and increase SNR [21]. At least two *b*-values are needed in order to calculate the corresponding apparent diffusion coefficient (ADC) map for image interpretation and disease response assessment [22]. The lowest *b*-value should be at least 50 s/mm^2^ in order to reduce perfusion-related signals, while a high *b*-value in the range between 800 and 1,000 s/mm^2^ is recommended [13, 21, 22]. The use of a single diffusion encoding direction with simultaneous application of gradients from all three axes can provide higher SNR by reducing echo times. It may also reduce the blurring that can arise when averaging across images acquired with multiple diffusion encoded directions that are prone to different eddy current induced distortions [23]. The values of ADC in tissues that have anisotropic diffusion (kidneys, and white matter) may, however, be biased with such diffusion encoding, though this is usually of secondary importance for WB-MRI examinations. WB DWI requires a relatively long (10–15 minutes) acquisition time [24, 25]. Obtaining fewer averages of the low *b*-value images, that have intrinsically higher SNR, is one measure for minimising acquisition times.

Coverage of the lung and brain can be incorporated with less than 2 minutes of scanning by using a single breath-hold, short echo-time GRE sequence for evaluation of lung parenchyma [26], and a fluid-attenuated inversion recovery TSE sequence for detection of focal brain lesions or brain oedema. While contrast media is not routinely used in WB-MRI studies, it may be useful for detecting brain metastases or meningeal disease [27]. Depending on clinical context, sequences to evaluate specific body regions can be added to a basic WB-MRI protocol [13]. For example, a post-contrast T1-weighted brain scan is recommended in Li–Fraumeni syndrome (LFS) [28]. For men, axial T2-weighted and DWI sequences targeted to the prostate can be added to provide an all-in-one prostate cancer (PC) staging examination [17, 29].

Following well-established practices for WB positron emission tomography (PET) and computed tomography (CT), WB-MRI acquisitions span from the vertex to mid-thigh. A distinction is that with the patient lying supine, the arms remain adducted. The acquisition should be extended to the feet when performed for diseases that frequently involve the extremities, such as neurofibromatosis [30], or as part of surveillance in cancer predisposition syndromes that favour soft-tissue tumours, such as LFS [31]. The acquisition of WB-MRI in patients affected by multiple myeloma should, instead, span from vertex to knees [11].

Both coronal and axial acquisitions have been used in WB-MRI [11, 17, 18]. The coronal orientation may permit the acquisition of fewer stations and thus reduce scan time. Coronal DWI scans are more likely, however, to suffer from distortion than axial DWI with the same field of view (FOV) and number of slices [32]. Axial acquisition has a further advantage in providing images that can be directly correlated with the conventional cross-sectional anatomy of other modalities. Slice thickness (SLT) should remain consistent across the different sequences to allow efficient image comparison and improve the readability of the examination. The choice of SLT remains somewhat variable; recommendations are for the use of contiguous slices with a thickness between 5 and 7 mm [11, 13].

WB-MRI can be performed on both 1.5 T and 3 T scanners, but 1.5 T may be preferred when patients have non-removable metallic prostheses. An advantage of 3 T scanners is seen in higher SNR, but the lower homogeneity of the static field can reduce the effectiveness of fat saturation leading to a variable SNR in DWI, and increases the risk of ‘phase-wrapping’ in DIXON scans [33].

The impact of magnetic field inhomogeneity is particularly evident where the superior and inferior limits of successive stations are seen adjacent to each other as happens when viewing the images in anatomic order or in maximum intensity projection (MIP). Attempting to deal with this problem by not shimming the different stations, by applying the same centre frequency to all stations or by limiting the superior-inferior coverage of each station [33] have largely been inadequate. Slice specific shimming [34], on the other hand, has provided consistent reduction, and is to be recommended where available.

Achieving WB coverage while optimising SNR requires the use of a large number of receiver coils. Given that most modern systems provide a fixed head-coil position, the patient is generally positioned headfirst in the head and neck array coil. In addition, a spine array coil and anterior body coil array(s) are necessary to cover the chest, abdomen and pelvis. If the examination is to be extended to the feet, a lower limb coil should be added in order to maintain a high SNR on the entire body [35]. To avoid time being lost on changeovers of the coils, all coils should be positioned and connected for the duration of the procedure. Because this effectively sandwiches the patient between arrays of coils, it is important to ensure they are comfortable and fully prepared to remain still for the duration of the examination. Preparation should include provision of accurate information about the examination, having the patient change into a single-use gown to avoid hidden metal objects and encouraging them to void their bladder prior to entering the scanner.

Image quality of the Dixon T1- and HASTE T2-weighted images may be improved if the chest and abdomen stations are obtained in breath-hold. The patient should be informed before the examination of the instructions and breath-hold duration. During the examination, they must be able to hear the instructions clearly and communications should be maintained between scans to ensure they are awake when the instructions are given.

### Post-processing and quantitative analysis

Where possible, ‘set-up and go’ acquisition and post-processing should be used to simplify and accelerate the operational workflow for WB-MRI and reduce the scope for operator errors. To facilitate image reading and reporting, we can unify (compose) the different stations of each sequence into single-stack of images with WB coverage. A series of MIPs of the high *b*-value DW images, rotating around the cranio-caudal axis, allow an ‘at-a-glance’ overview for disease assessment and lesion detection. Typically, these are displayed in inverse grey scale, thus providing an appearance similar to that of PET.

Relative fat fraction (rF%) maps have emerged as an objective image-based biomarker of disease [36]. Obtained from T1 GRE Dixon images, rF% can provide a quantitative assessment of the distribution of fat within the body, and of oncological relevance, describe bone marrow substitution [37]. The rF% maps are obtained by dividing the signal intensity of the fat-only images (F) by the sum of the fat-only and water-only (W) images:

$$\mathrm{rF}\%=\frac{100\times F}{F+W}$$

ADC maps are useful for classifying findings and response to therapy [11, 13]. ADC is usually calculated with a monoexponential fitting to the signal intensities in the different *b*-value DWI images:

$$\mathrm{ADC}=-\ln\frac{\left(\frac{S_{\mathrm{high}}}{S_{\mathrm{low}}}\right)}{(b_{\mathrm{high}}-b_{\mathrm{low}})},$$

where *S_high_* and *S_low_* are the signal intensities in the high and low *b*-value images (*b_high_* and *b_low_*), respectively [38].

The large number of images produced in each WB-MRI examination complicates extracting the quantitative information provided via rF% and ADC maps. This necessitates considerable time for reading and reporting, and can increase the risk of misinterpretations. Several semi-automatic segmentation techniques have been developed to assist in distinguishing malignant lesions from healthy tissue and benign findings [37, 39]. A recently proposed tool for WB DWI segmentation combines application of thresholds and manual editing to provide a relatively efficient segmentation of a distributed lesion load (Figure 1). In evaluation of bone marrow and of bone metastases [22, 40] it has shown very good repeatability [41, 42] suggesting utility in clinical practice.

Despite considerable progress, WB-MRI still faces a number of technical and practical challenges. Several of these are tied to the quality of the static and radiofrequency magnetic field homogeneities that are available [33]. When attempting to obtain large anatomical coverage in a single scanning station, the static field inhomogeneity can yield distortions that can disrupt anatomy, for example, creating a ‘broken spine’ artefact between stations on DWI scans, and producing water-fat swaps in the Dixon technique. Radio-frequency inhomogeneities are associated with the use of large numbers of small coils as well as being a function of patient size and composition that can lead to inhomogeneous signal intensity in deep structures or between imaging stations. Both these issues become more pronounced on moving from 1.5T to 3.0T, which is otherwise favourable to reducing the number of averages necessary (and thus scan time) or pursuing higher spatial resolution. With advanced shimming techniques [43], these can be addressed to some extent, though the shimming may add notably to the examination duration. Practical issues include a greater sense of being in a close space because of the coverage of the patient with coils, and the need to cope with a long, noisy examination. Nonetheless, when informed well, patients appear to appreciate WB-MRI as an effective imaging technique and see it favourably in comparison to other imaging procedures [44].

## Clinical Applications

WB-MRI has entered a clinical domain dominated by bone scan (BS), (CT), PET and the combination—PET/CT. In WB-MRI, morphological images are produced with a choice of contrasts being available, including DWI associated with changes in the cell density, and T1- and T2-weightings that largely reflect fat and water and fat content, while PET contrast derives from metabolic activity of the administered radioactive tracer. Consequently, differences in sensitivity between WB-MRI and PET are typically attributed to mismatches between hypercellularity or chemical content, and altered metabolism in the chemical pathway targeted by the specific PET tracer. WB-MRI examination times tend to be longer than those of PET, but the examination does not necessitate injection of a radioactive tracer, nor the wait for the tracer to achieve adequate biodistribution for imaging.

### Multiple myeloma

Britain’s National Institute for Health and Care Excellence guidance recommends WB-MRI as first-line imaging for suspected myeloma and include it along with fluorodeoxyglucose (FDG)-PET/CT and spine MRI for monitoring in case of serological relapse or disease progression [45]. The Oxford Centre of Evidence-based Medicine similarly recommends WB-MRI for staging all forms of multiple myeloma [10] while the British Society for Haematology recommends WB-MRI for therapy monitoring in multiple myeloma patients [46]. Reflecting the application to therapy monitoring, the Myeloma Response Assessment and Diagnosis System (MY-RADS) was published in 2019 to promote standardisation and reduce variation in acquisition, interpretation and reporting of WB-MRI. MY-RADS describes a core clinical imaging protocol with extension for advanced assessment, a structured reporting template and guidance on defining response assessment categories that incorporates ADC measurements [11].

### Prostate cancer

At initial staging for unfavourable intermediate risk (Gleason Score 4+3) and high-risk PC patients, European Association of Urology (EAU) guidelines recommend at least cross-sectional abdominopelvic imaging and a BS [12]. Similarly, EAU and National Comprehensive Cancer Network (NCCN) guidelines recommend systemic staging with CT and BS for patients with advanced PC (APC). WB-MRI and PET have entered the recent American Society of Clinical Oncology (ASCO) guidelines, where they are indicated for screening where conventional imaging is negative or equivocal [47]. Multiple studies have recognised that MRI is more sensitive than CT and BS for the detection of bone metastases [48, 49]. For this reason, an ‘all-in-one’ approach (Table 2) that combines multiparametric MRI of the prostate with a WB-MRI study is attractive for local and systemic staging [13, 50].

**Figure 2.**
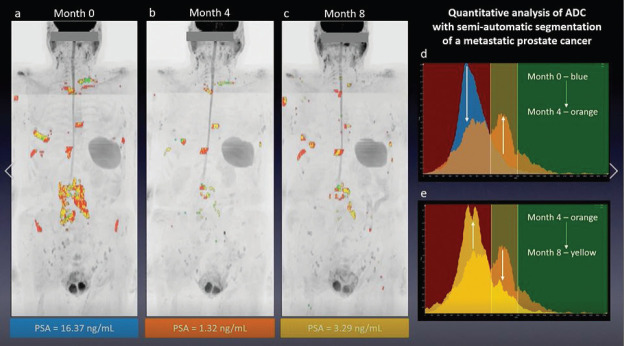
Metastatic PC staging with WB-MRI. A 70-year-old male with high-risk PC (PSA 28 ng/mL, Gleason score (GS) 4+4) seen in (a): coronal inverted grey-scale MIP of b-900 diffusion-weighted images and (b–d): axial b-900 diffusion-weighted images b-900 images from WB-MRI performed for staging. In the ‘at-a-glance’ disease assessment facilitated by the MIP, WB-MRI shows a clinical pattern of type N1 M1b, involving (b): regional lymph node metastasis (c): a non-regional lymph node metastasis and (d): bone marrow disease.

For APC, the ASCO guidelines consider WB-MRI, PET/CT and PET/MRI as alternatives [47]. As prostate membrane specific antigen (PSMA) PET/CT may not inform on tumour viability during androgen receptor inhibition [51], the option of pairing it with FDG-PET has been suggested [52], but bears a significant radiation exposure compared to WB-MRI. Although some studies have shown very good performance of Choline PET for both local and distant disease in staging (e.g. [53]), a 2018 review of the literature suggests it does not perform so well in all circumstances [54]. The Advanced Prostate Cancer Consensus Conference recognises the limitations of BS and CT for bone assessment, and highlights that the flare phenomenon can interfere with the therapy response assessment [55, 56]. Notably, WB-MRI is not affected by this phenomenon and has clear value in the detection of skeletal related events such as spinal cord compression and fractures. Moreover, for up to 30% of patients with metastatic castration resistant PC, there is a radiographic progression without clinical/prostate-specific antigen (PSA) progression [57]. Instead, WB-MRI is increasingly used for APC therapy monitoring (Figure 2) and for patients experiencing biochemical recurrence that, in case of oligometastatic disease (one to five oligometastatic lesions), are potentially candidates for local therapy [58]. Evidence from a randomised trial shows that the stereotactic ablative radiotherapy is associated with an improvement in overall survival in patients with oligometastatic PC [59].

Lymph nodes are another common site of PC metastasis and are thus a concern both in staging and in oligoprogression. In a 2016 study by Larbi et al [60], only one-third of enlarged lymph nodes seen on WB-MRI in metastatic prostate cancer patients were within the the regions recommended for external beam radiation therapy or extended lymph node resection, illustrating the importance of whole-body lymph node assessment. MRI and CT, dependent on morphological assessment of nodal disease, have similar but unfortunately limited levels of performance in detection of metastatic nodal detection [61]. Several PET tracers, including 2-deoxy-2-(^18^F)fluoro-D-glucose, sodium (^18^F)fluoride, (^11^C)choline and (^18^F)choline have been found to perform comparably or worse than MRI and CT for nodal disease, but recent preliminary reports suggest that PET/CT with some (PSMA)-based tracers may permit greater sensitivity without sacrificing specificity [62, 63]. Nonetheless in light of the above-mentioned performance for bone metastases, WB-MRI remains attractive alone or in combination with PSMA PET as the clinical significance of the sub-centimetre nodes that are poorly seen with WB-MRI remains to be established. Moreover, WB-MRI in APC is able to better reflect the heterogeneity of disease [13].

Standards for image acquisition, interpretation and reporting for clinical care and clinical trials involving APC patients have recently been defined in the Metastasis Reporting and Data System for Prostate Cancer (MET-RADS-P) guidelines [13]. For reporting, these guidelines consider 14 anatomical regions in a baseline template and response assessment criteria for following disease evolution in follow-up.

### Melanoma

The German Dermatological Society and the Dermatologic Cooperative Oncology Group [14, 15] suggest the use of WB-MRI as a valid alternative to contrast-enhanced CT or PET/CT for follow-up of advanced melanoma (stage III or higher) and this is also the recommendation in Swiss guidelines for the treatment and follow-up of cutaneous melanoma [14]. WB-MRI has demonstrated comparable diagnostic performance in the detection of extracranial metastases from melanoma and other tumours in multiple body regions [64, 65]. Lung parenchyma is difficult to study with the conventional T1-, T2- and diffusion-weighted images. Breath-hold, short echo-time, GRE images have been reported to perform well in demonstrating pulmonary lesions greater than 5 mm [66]. Taking advantage of techniques to improve resolution [64], detection of smaller lesions can be expected; and if negative, low-dose CT of the lungs considered [26]. The study of liver, which has always created concerns for the absence of contrast agent, is performed with DWI and allows detection, even of small metastases, with good sensitivity [67].

### Breast cancer

Currently, international guidelines are mixed as to the which WB imaging techniques to apply and under what conditions (staging, monitoring, recurrence) in the evaluation of breast cancer (BC) patients (see [68] for a summary). CT, BS and FDG-PET dominate European Society for Medical Oncology (ESMO), Royal College of Radiologists (UK) and North American NCCN recommendations for the contexts they cover, with conventional MRI and ultrasound being alternatives for abdominal imaging where appropriate [69–71]. In a direct comparison, the SKELETA Trial [72] showed that WB-MRI provides higher sensitivity for bone metastases arising from breast and PC than BS, single photon emission computed tomography (SPECT) and SPECT-CT (91% versus 62%, 74% and 85%, respectively), and nearly identical sensitivity to ^19^F-NaF PET-CT (93%) consistent with previous bimodality comparisons [73, 74].

The recent Response Evaluation of Cancer Therapeutics (RESPECT) study [75] monitored treatment response in a cohort of 44 women with metastatic BC using bone scintigraphy, CT and WB-MRI at 12-week intervals. Amongst the 33 patients who arrived at radiological progression during the study, all were seen with WB-MRI at time of first appearance of progression, but only 11 of these were evident on the CT of the corresponding time-point. In the 26 patients whose progression involved bone, just 13 cases were considered in progression on the bone scintigraphy at the time of radiological progression. This strongly suggests the suitability of WB-MRI for response assessment in systemic anticancer therapy.

As BC is the second most common cause of brain metastases [76], contrast-enhanced brain MRI should be considered in these patients as the sensitivity has been seen to exceed those of CT and FDG-PET [77]. Liver metastases are also relatively common in BC patients. In the imaging of liver metastases is DWI well-established as outperforming both CT and PET [77, 78].

A subgroup of BC patients for whom WB-MRI is seeing increasing use, is that of young BC patients for whom serial treatment monitoring is necessary. This use of WB-MRI is driven by growing awareness of radiation induced risks, especially for those under 35 years of age [79].

WB-MRI is also establishing an important role in the care of pregnant women with BC [25, 80] where systemic staging is mandatory in preparation for childbirth-based therapies. As a technique without ionising radiation and requiring no contrast agent, it is more appropriate than CT or BS for staging and therapy monitoring in these patients.

### Ovarian cancer

For staging and follow-up of ovarian cancer patients, the European Society of Urogenital Radiology guidelines recommend contrast-enhanced chest-abdomen-pelvis CT [81]. Evidence shows that WB-MRI has higher diagnostic accuracy than CT in determining the malignant nature of ovarian masses [82], as well as for assessing the involvement of mesentery, para-aortic lymph nodes, large bowel and sigmoid-rectum [83]. Furthermore, quantitative analysis using ADC measurements has proven robust to detect early microstructural changes occurring in response to advanced epithelial ovarian cancer [84]. WB-MRI has also been shown to be more accurate than both CT and FDG-PET in the staging of peritoneal lesions (91% versus 75% and 71% respectively), and both WB-MRI and FDG-PET to be more accurate than CT in retroperitoneal lymphadenopathy detection (respectively, 87% and 87% versus 71%) [85]. These promising results suggest that WB-MRI could have a role in the management of ovarian cancer patients.

### Lymphoma

**Figure 3.**
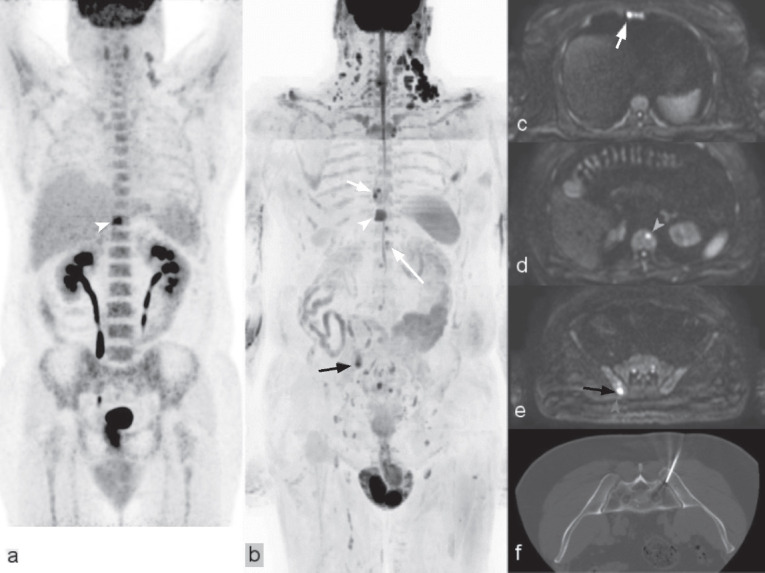
Bone lesions in lymphoma. A 51-year-old male with follicular lymphoma (G1-G2). (a): ^18^FDG-PET for distant staging to have a single bone metastasis of the T10 vertebra (white arrowhead). At WB-MRI 1 month later, oligometastatic disease with multiple bone lesions was evident in (b): the inverted grey scale MIP of the b-900 diffusion weighted images; with corresponding hyperintensities being seen in the axial source images (c–e). In addition to the T10 metastasis (white arrowhead in a and b), a sternal metastasis (smaller white arrow in b and c), a second thoracic vertebral lesion (longer white arrow in b and d) and an iliac lesion (black arrow in b and e). Metastasis in the right iliac bone was confirmed by CT-guided biopsy (f).

Owing to its high accuracy, FDG-PET/CT is the recommended imaging technique for the staging and follow-up of most common lymphomas (e.g. follicular lymphoma and Hodgkin’s lymphoma) [86]. It has been demonstrated, however, that WB-MRI shows higher sensitivity (94%) for lymphoma detection in patients with variable FDG avidity lymphoma subtypes than FDG-PET/CT (61%) and contrast-enhanced CT (71%) [87] (Figure 3). Furthermore, WB-MRI use is emerging in young patients (<35 years) [79] and pregnant women (lymphoma occurring in 1 pregnancy in 1,000) [88] to minimise exposure to ionising radiation.

### Lung cancer

NCCN guidelines recommend the staging of lung cancer with PET/CT and/or total body CT, with the addition of an MRI examination of the brain when needed [89]. The Streamline L study has shown that entirely WB-MRI-based pathways are viable in replacement for standard pathways and require shorter staging time [90]. In lung cancer patients for whom a brain MRI is necessary, integration of this examination with WB-MRI to allow tumor-node-metastasis (TNM) staging in a single sitting is an obvious step for efficiency.

### Colorectal cancer

ESMO and NCCN guidelines both recommend an abdomen-pelvis CT for colon cancer staging, and a chest-abdomen-pelvis CT together with a rectal MRI for rectal cancer staging [91, 92]. The Streamline C study, however, found that WB-MRI-based pathways are a viable replacement for standard pathways in staging patients with colon cancer [93]. Considering this, the role of WB-MRI in the management of both colon and rectal cancer patients can be expected to expand. Moreover, combining WB-MRI systemic staging with rectal MRI for local staging could be of interest as a one-stop-shop for TNM staging of rectal cancer in a single session.

### Cancer screening

Due to its spatial resolution and high sensitivity, along with its non-invasive nature and the absence of ionising radiation [94], WB-MRI has emerged as the imaging technique of choice for cancer screening in subjects with cancer predisposition syndromes [28, 31, 95, 96].

According to the American Association for Cancer Research (AACR), as well as a multicentre cohort review involving 13 cohorts and 578 participants, an annual WB-MRI is recommended for patients with LFS as a screening examination [28, 31]. The exam should be complemented with a contrast-enhanced brain MRI and, in women, with a contrast-enhanced breast MRI as well.

For constitutional mismatch repair deficiency (CMMR-D) syndrome, an annual WB-MRI examination from the age of 6 years has been recommended in a consensus statement by the Care for CMMRD Consortium and the International Biallelic Mismatch Repair Deficiency Consortium [95]. For hereditary paraganglioma-pheochromocytoma, on the other hand, the AACR has recommended biennial WB-MRI [96].

The application of WB-MRI to oncological screening in the general population is still a matter of debate. Several reports have demonstrated the use of WB-MRI in asymptomatic individuals [27, 97, 98–106], the largest of which includes 30,000 subjects [107]. Although the methods and results of the studies are too heterogeneous to draw unequivocal conclusions, enough tumours have been diagnosed in asymptomatic subjects to generate clinical interest in the technique and to motivate further research.

## Conclusions

Thanks to a series of improvements in scanner hardware and imaging technique, it is possible to achieve in clinical routine what 20 years ago seemed impossible: a WB-MRI examination that provides a core clinical protocol for WB imaging in little more than half an hour. Continuing developments in imaging strategy and artificial intelligence hold the promise of further improvements and are likely to expand the use cases of WB-MRI in oncology. Existing guidelines demonstrate the central role of WB-MRI examinations with DWI in some oncology applications, but further efforts to standardise acquisition and reporting are warranted to reduce the variation in diagnostic performance.

## Trial registration

Not applicable.

## Conflicts of interest

Dr Summers is co-owner of QMRI Tech, a company that provides support for medical physics activities and MRI research.

The other authors declare that they have no conflicts of interest.

## Funding

This work was funded by FIEO-CCM, and partially supported by the Italian Ministry of Health with ‘Ricerca Corrente’ and 5x1000 funds. The funding bodies did not participate in the planning, design or execution of the study.

## Figures and Tables

**Figure/Video 1.**
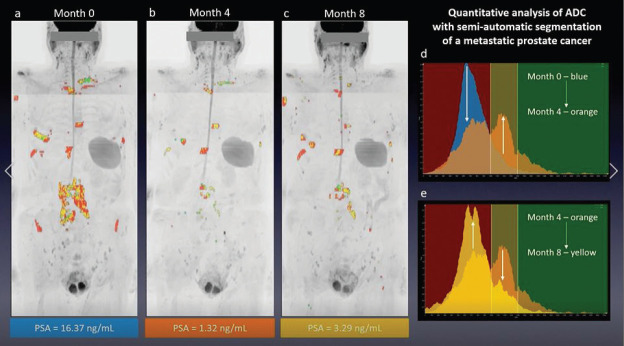
Illustration of the quantitative analysis of ADC via semi-automated segmentation in a patient with metastatic PC. To view this video, click here https://ecancer.org/journal/14/1064-whole-body-magnetic-resonance-imaging-technique-guidelines-and-key-applications. Male with hormone-sensitive metastatic PC seen at (a): baseline, (b): 4 month follow-up and (c): 8 month follow-up examinations during hormonal therapy. Overlaid on inverted grey-scale MIPs from b-900 diffusion weighted images are colours representing three ranges of ADC values. Based on MET-RADS-P guidelines [13], the user-defined thresholds (1,000 and 1,400 μm^2^/s) define ranges corresponding to: active disease (red) < 1,000 μm^2^/s < normal bone/responding lesion (yellow) < 1,400 μm^2^/s < treated disease/necrotic tissue (green) measured in the bone metastases. These ranges also serve to define the coloured backdrop to comparisons of ADC histograms between successive examinations in (d) and (e). (d): The peak of the (blue) histogram at baseline falls in the active disease range (red band), while the (orange) histogram at first follow-up shows two peaks. The right-most peak, falling in the likely-response range (yellow band) indicating according to the MET-RADS-P guidelines [13], together with a reduction in PSA, this could suggest a positive therapeutic response, but the left-hand peak indicates a residual component of disease is still active. At the second follow-up (yellow histogram), (e): the distribution has returned to the active disease range (red band) and the PSA having increased, is compatible with a predominance of active metastatic disease.

**Table 1.** Sequence components for WB-MRI examinations.

Sequence description		Multiple myeloma [11] (MY-RADS)	Metastatic prostate cancer [13] (MET-RADS-P)	Suggested protocols			
				Breast cancer	Ovarian cancer	Lymphoma	Screening
1	Whole spine—sagittal, T1W TSE^c^, 4–5 mm SLT	Core	Core	Yes	Yes	No	No
2	Whole spine—sagittal T2W TSE STIR (preferred) or fat suppressed, 4–5 mm SLT	Core	Core	Yes	Yes	Yes	Yes
3	WB—axial, T1W GRE Dixon^c^, 5 mm SLT^d^Fat image reconstructions mandatory	Core vertex to knees	Core^b^ vertex to mid-thighs	Yes vertex to mid-thighs	Yes vertex to mid-thighs	Yes vertex to mid-thighs	Yes vertex to mid-thighs
4	WB—axial, diffusion weighted STIR, 5–7 mm SLT^d^ b50–100 s/mm^2^ and b800–1,000 s/mm^2^^e^ ADC calculation by mono-exponential data fitting Coronal b800–1,000 reconstruction^f^ 3D-MIP reconstructions of highest b-value images^g^	Core^a^ vertex to knees	Core skull base to mid-thighs	Yes skull base to mid-thighs	Yes skull base to mid-thighs	Yes skull base to mid-thighs	Yes skull base to mid-thighs
5	WB—axial, T2W TSE without fat- suppression, 5 mm SLT^d^	CA. vertex to knees	CA vertex to mid-thighs	Yes vertex to mid-thighs	Yes vertex to mid-thighs	Yes vertex to mid-thighs	Yes vertex to mid-thighs
6	Lung: 3D T1W GRE volume interpolated breath-hold examination <3 mm SLT, with short TE (<1.5 ms)			Yes	Yes	Yes	Yes
7	Regional assessments—if clinically needed	Symptomatic or known sites outside standard FOV	No	Brain: T1W axial, T2W axial, T2W coronal, post-contrast 3D T1W GRE at 0.6 mm^a^	Abdomen: T2W coronal without fat-suppression 5 mm^a^ contiguous		

ADC, Apparent diffusion coefficient; CA, Comprehensive assessment; FOV, Field of view; GRE, Gradient echo; MIP, Maximum intensity projection; SLT, Slice thickness; STIR, Short tau inversion recovery; TE, Echo time; TSE, Turbo spin echo; W, Weighted; 3D, Three-dimensional; BC, Breast Cancer; METastasis Reporting and Data System for Prostate Cancer, MET-RADS-P; Myeloma Response Assessment and Diagnosis System, MY-RADS; PC, Prostate Cancer

a Coronal acquisition accepted under MY-RADS

b Coronal acquisition with 2 mm SLT accepted under MET-RADS-P

c Alternatively, a 3D T1W TSE allowing multiplanar reformatting may be performed

d Suggest axial with common SLT across WB images to facilitate image review

e Add b500–600 s/mm^2^ for CA

f b800–1,000 s/mm^2^ DWI images from all stations grouped and reconstructed as 5 mm contiguous, two-dimensional coronal slices

g 3D MIP images, displayed rotating around cranio-caudal axis, using an inverted greyscale

**Table 2.** ‘All-in-one’ protocol with WB-MRI for PC staging.

Sequence	Plane and coverage	Parameters	Assessment
T1W TSE	Sagittal whole-spine	4–5 mm^a^	Bone metastases
T1W GRE Dixon	Axial WB	5 mm^a^	Bone and lymph nodes metastases
DWI	Axial WB	5–7mm^a^ 2 *b*-values: low: 50–100 s/mm^2^ high: 800–1,000 s/mm^2^	Bone and lymph nodes metastases
T2W TSE	Axial and sagittal prostate	3 mm^a^ small FOV	Prostate
DWI	Axial prostate	3 mm^a^ small FOV	Prostate

W, Weighted; TSE, Turbo spin echo; GRE, Gradient echo; DWI, Diffusion weighted imaging; FOV, Field of view

a slice thickness
